# Multiple imputation with competing risk outcomes

**DOI:** 10.1007/s00180-024-01518-w

**Published:** 2024-06-26

**Authors:** Peter C. Austin

**Affiliations:** 1 Institute for Clinical Evaluative Sciences (ICES), V106, 2075 Bayview Avenue, Toronto, ON M4N 3M5 Canada; 2 Institute of Health Policy, Management and Evaluation, University of Toronto, Toronto, ON Canada; 3 Sunnybrook Research Institute, Toronto, ON Canada

**Keywords:** Competing risks, Survival analysis, Missing data, Multiple imputation, Monte Carlo simulations

## Abstract

In time-to-event analyses, a competing risk is an event whose occurrence precludes the occurrence of the event of interest. Settings with competing risks occur frequently in clinical research. Missing data, which is a common problem in research, occurs when the value of a variable is recorded for some, but not all, records in the dataset. Multiple Imputation (MI) is a popular method to address the presence of missing data. MI uses an imputation model to generate M (M > 1) values for each variable that is missing, resulting in the creation of M complete datasets. A popular algorithm for imputing missing data is multivariate imputation using chained equations (MICE). We used a complex simulation design with covariates and missing data patterns reflective of patients hospitalized with acute myocardial infarction (AMI) to compare three strategies for imputing missing predictor variables when the analysis model is a cause-specific hazard when there were three different event types. We compared two MICE-based strategies that differed according to which cause-specific cumulative hazard functions were included in the imputation models (the three cause-specific cumulative hazard functions vs. only the cause-specific cumulative hazard function for the primary outcome) with the use of the substantive model compatible fully conditional specification (SMCFCS) algorithm. While no strategy had consistently superior performance compared to the other strategies, SMCFCS may be the preferred strategy. We illustrated the application of the strategies using a case study of patients hospitalized with AMI.

**Supplementary Information:**

The online version contains supplementary material available at 10.1007/s00180-024-01518-w.

## Background

The occurrence of missing data is common in applied research. Missing data occurs when the value of a variable is recorded for some, but not all, records in the dataset. Multiple Imputation (MI) is a method for addressing the presence of missing data that entails the creation of M (M > 1) complete datasets, in which missing values have been replaced by plausible values generated using an imputation model (Rubin 1987). A separate statistical analysis is conducted in each of the complete datasets, and the results are pooled across the complete samples.

Fully conditional specification (FCS) is an MI method which specifies multivariate models through conditional distributions. Multivariate imputation using chained equations (MICE) is a popular algorithm for implementing FCS, with each variable imputed conditional on all other variables (van Buuren 2007, 2018; van Buuren and Groothuis-Oudshoorn 2011; White et al. 2011). It is important that the imputation model for a given variable includes, as a predictor, the outcome for the final analysis model that is of scientific interest (Moons et al. 2006; Sterne et al. 2009; White et al. 2011). Thus, if the outcome in the analysis model is continuous (e.g., blood pressure) or binary (e.g., treatment success), that variable should be included as a predictor variable in the imputation models. Similarly, if the outcome in the analysis model is time-to-event in nature, this variable should be included as a predictor variable in the imputation models. In the presence of censoring, the time-to-event outcome has two components: (i) the observed survival time denoting the time to the occurrence of the outcome of interest or the time to censoring; (ii) a binary indicator variable denoting whether the subject experienced the event of interest or whether they were censored. Both these components should be included in the imputation models (White and Royston 2009). There are different ways of including the time component: (i) as the observed survival time; (ii) the logarithm of the observed survival time; (iii) as the empirical cumulative hazard function at the observed survival time. White and Royston showed that the latter is preferable (White and Royston 2009).

Time-to-event or survival outcomes are common in medical and epidemiological research, with a common example being time to death. A competing risk is an event whose occurrence precludes the occurrence of the primary event of interest. Thus, if the outcome is time to cardiovascular death, non-cardiovascular death would be a competing risk, since those who die of non-cardiovascular causes are no longer at risk of dying of cardiovascular causes (Austin et al. 2016). If there are K different event types, then there are K cumulative hazard functions of interest: the K cause-specific cumulative hazard functions. There is limited research on how to incorporate the outcome of the analysis model into imputation models when using MICE and the analysis model of scientific interest is a cause-specific hazard model (Bartlett and Taylor 2016; Bonneville et al. 2022). When using MICE, options include: (i) including only the cumulative hazard function for the primary outcome of interest along with its event indicator variable; (ii) including the K cumulative hazard functions for the K event types along with the event type variable. Note that with competing risks there exist several regression models of interest, including the Fine-Gray subdistribution hazard model for the cumulative incidence function. In the current study we focus on the cause-specific hazard model due to its widespread use in medical and epidemiological research. As noted above, this issue was addressed using simulations by two previous sets of authors (Bartlett and Taylor 2016; Bonneville et al. 2022). However, the simulation designs were such that there were only two types of events, the analysis model had only two or three variables, and there was only one pattern of missingness. We wanted to evaluate the performance of the different imputation strategies using a more complex set of simulations that are more reflective of analyses conducted in clinical research.

The objective of the current paper was to compare the relative performance of different strategies for including the outcome of the analysis model in the imputation model in the presence of competing risks. The paper is structured as follows. In Sect. 2, we describe the design of a series of Monte Carlo simulations to address this issue. These simulations are based upon an analysis of patients hospitalized with acute myocardial infarction (AMI), so that the simulations reflect a clinically realistic setting. In Sect. 3, we report the results of these simulations. In Sect. 4, we provide a case study illustrating the application of the different strategies for estimating three cause-specific hazard models in patients hospitalized with AMI. Finally, in Sect. 5, we summarize our findings and place them in the context of the existing literature.

## Methods

We conducted a series of Monte Carlo simulations to compare the performance of different strategies for incorporating the outcome of the analysis model in imputation models when the analysis model of scientific interest is a cause-specific hazard model in the presence of competing risks. We evaluated the performance of each strategy in the setting of making inferences about the regression coefficients in a multivariable cause-specific hazard model with 10 covariates, five of which were continuous and five of which were binary. There was one primary event type and two competing risks. The design of the simulations was informed by empirical analyses of patients hospitalized with AMI. The data and the design of the simulations were similar to those used in a recent study that examined the performance of MI when the prevalence of missing data was very high (Austin and van Buuren 2022).

### Data and empirical analyses to provide parameters for the Monte Carlo simulations

The data consisted of 19,395 patients hospitalized with a diagnosis of AMI. In the current study, the outcome was time to cause-specific death, with three types of events being considered: death due to cardiovascular causes, death due to cancer, and death due to other causes. The primary analysis model of scientific interest was the cause-specific hazard model for cardiovascular death, with the two other cause-specific hazard models being of secondary interest. In each model, the cause-specific hazard of interest was regressed on 10 predictor variables: age, systolic blood pressure, heart rate, hemoglobin, cholesterol, sex, angina, diabetes, history of previous AMI, and current smoker. The first five are continuous, while the last five are binary.

Time of death, cause of death, age, and sex were not subject to missing data as they can be ascertained through linkages to provincial health insurance registries. Overall, 48.1% of patients had missing data for at least one variable. Amongst the 9325 subjects with missing data, there were 67 patterns of missing data. The prevalence of missing data for those 8 variables that were subject to missingness were: 0.9% (systolic blood pressure), 1.2% (heart rate), 1.2% (hemoglobin), 40.5% (cholesterol), 1.4% (angina), 0.4% (diabetes), 1.6% (previous AMI), and 13.4% (current smoker). A more detailed description of these data and how imputation was used to impute missing data in these empirical data are described in the original study (Austin and van Buuren 2022). For each of the eight variables subject to missingness, the imputation model included: (i) the other nine predictor variables from the scientific model; (ii) the three cumulative cause-specific hazard functions and the three event indicator variables; (iii) the interactions between each of the three cumulative cause-specific hazard functions and the nine predictor variables from the scientific model. Based on the recommendation of White and colleagues that the number of imputed complete datasets equal the percentage of subjects for whom there is any missing data, we created 48 complete datasets given that 48% of patients were subject to missing data (White et al. 2011).

We conducted the following analyses in the 48 complete datasets: (i) estimated the coefficients of the three analysis models in which the cause-specific hazard was regressed on the 10 predictor variables; (ii) estimated the variance–covariance matrix of the ten predictor variables (in doing so we treated the five binary variables as continuous variables); (iii) estimating the means and prevalence of each of the ten predictor variables. For each analysis the estimated parameters were pooled using Rubin’s Rules (Rubin 1987). The pooled estimate of the variance–covariance matrix was used to simulate predictor covariates, while the three estimated cause-specific hazard models was used to generate outcomes. Let ${{\varvec{\upbeta}}}_{{{\text{cardiovascular}}}}$, ${{\varvec{\upbeta}}}_{{{\text{cancer}}}}$, and ${{\varvec{\upbeta}}}_{{{\text{other}}}}$ denote the vectors of estimated regression coefficients for the three cause-specific hazard models that were obtained using Rubin’s Rules. The estimates of the regression coefficients for the three cause-specific hazard models are listed in Table 1.

**Table 1:** Regression coefficients for the three cause-specific hazard models

Variable	${{\varvec{\upbeta}}}_{{{\text{cardiovascular}}}}$	${{\varvec{\upbeta}}}_{{{\text{cancer}}}}$	${{\varvec{\upbeta}}}_{{{\text{other}}}}$
Age	0.075	0.062	0.077
Systolic blood pressure	− 0.007	− 0.001	− 0.004
Heart rate	0.009	0.007	0.009
Hemoglobin	− 0.008	− 0.016	− 0.016
Cholesterol	− 0.051	− 0.042	− 0.073
Female	− 0.045	− 0.343	− 0.122
Angina	0.147	− 0.203	− 0.038
Diabetes	0.297	0.057	0.893
Previous AMI	0.398	0.0154	0.297
Current smoker	0.226	0.584	0.308

### Factors in the Monte Carlo simulations

We allowed two factors to vary in our simulations: p_missing_ (proportion of records that had missing data) and the rate parameters for the three cause-specific baseline hazard functions ($\lambda_{{{\text{cardiovascular}}}}$,$\lambda_{{{\text{cancer}}}}$,$\lambda_{{{\text{other}}}}$). These denote the rates when all covariates were set equal to zero. The former took 6 values: from 0.1 to 0.6 in increments of 0.1. For the latter, we considered five sets of rate parameters: {$(\lambda_{{{\text{cardiovascular}}}} = 1,\;\lambda_{{{\text{cancer}}}} = 1/3,\;\lambda_{{{\text{other}}}} = 1/3)$,

$(\lambda_{{{\text{cardiovascular}}}} = 1,\;\lambda_{{{\text{cancer}}}} = 1/2,\;\lambda_{{{\text{other}}}} = 1/2)$, $(\lambda_{{{\text{cardiovascular}}}} = 1,\;\lambda_{{{\text{cancer}}}} = 1,\;\lambda_{{{\text{other}}}} = 1)$, $(\lambda_{{{\text{cardiovascular}}}} = 1,\;\lambda_{{{\text{cancer}}}} = 2,\;\lambda_{{{\text{other}}}} = 2)$, $(\lambda_{{{\text{cardiovascular}}}} = 1,\;\lambda_{{{\text{cancer}}}} = 3,\;\lambda_{{{\text{other}}}} = 3)$}. In the third set of rate parameters, the three events occur at an equal rate for a subject whose covariates are all equal to zero, while in the other four sets of rate parameters, the rate at which cardiovascular events occur for a subject whose covariates are all equal to zero is either higher or lower than the rate at which the other two types of events occur. We set the $\lambda_{{{\text{cardiovascular}}}}$ to equal 1 and chose the other rate parameters to be either smaller or larger than the rate parameter for cardiovascular death. The decision to set the rate parameters for cancer deaths and other deaths to be equal to one another was made for pragmatic reasons due to the limited number of scenarios that we were able to examine. We used a full factorial design and thus considered 30 different scenarios. The simulations generated a super-population from which a random sample of size 1,000 will be sampled in each of the iterations of the simulations.

### Monte Carlo simulations: Simulating a super-population

We designed a series of Monte Carlo simulations to compare the performance of strategies for imputing missing data in the setting of competing risks. We used the means/prevalences, the variance–covariance matrix, and the regression coefficients for the three cause-specific hazard models estimated above to generate a super-population that was similar to the empirical data described above.

For each subject in a super-population of size 1,000,000 we simulated 10 predictor variables from a multivariate normal distribution with mean vector and variance–covariance matrix equal to those estimated above. The first five variables were retained as continuous while the last five variables were dichotomized. These last five variables were dichotomized using a threshold selected so that the prevalence of the resultant binary variable was equal to the prevalence of the corresponding binary variable estimated above. Thus, in the super-population, the 10 simulated predictor variables had a multivariate distribution that was similar to that observed in the empirical AMI sample. For ease of description, we refer to each of the 10 simulated predictor variables using the name of the variable in the empirical data that that simulated variable was intended to mimic.

We then generated a time-to-event outcome for each subject in the super-population. To do so, we used Beyersmann’s algorithm for simulating competing risk outcomes (Beyersmann et al. 2009). We assumed that the underlying baseline hazard function for each of the three event types was constant with rate parameters $\lambda_{{{\text{cardiovascular}}}}$, $\lambda_{{{\text{cancer}}}}$, and $\lambda_{{{\text{other}}}}$. If each of the three types of events follows an exponential distribution with hazard function $h(t) = \lambda$, the occurrence of all-cause mortality (cardiovascular death or cancer death or death due to other causes) follows an exponential distribution with hazard function $\lambda_{{{\text{cardiovascular}}}} + \lambda_{{{\text{cancer}}}} + \lambda_{{{\text{other}}}}$. For each subject, we computed the linear predictor for the three cause-specific hazard models: ${\mathbf{X\beta }}_{{{\text{cardiovascular}}}}$, ${\mathbf{X\beta }}_{{{\text{cancer}}}}$, and ${\mathbf{X\beta }}_{{{\text{other}}}}$, where ${\mathbf{X}}$ denotes the vector of 10 predictor variables. Using Bender’s algorithm we generated an event time $T = \frac{ - \log (u)}{{\lambda_{{{\text{cardiovascular}}}} \exp ({\mathbf{X\beta }}_{{{\text{cardiovascular}}}} ) + \lambda_{{{\text{cancer}}}} \exp ({\mathbf{X\beta }}_{{{\text{cancer}}}} ) + \lambda_{{{\text{other}}}} \exp ({\mathbf{X\beta }}_{{{\text{other}}}} )}}$, where u ~ U(0,1) (Bender et al. 2005). We then simulated an event type such that cardiovascular events, cancer events, and other events occurred with probability $\frac{{\lambda_{{{\text{cardiovascular}}}} \exp ({\mathbf{X\beta }}_{{{\text{cardiovascular}}}} )}}{{\lambda_{{{\text{cardiovascular}}}} \exp ({\mathbf{X\beta }}_{{{\text{cardiovascular}}}} ) + \lambda_{{{\text{cancer}}}} \exp ({\mathbf{X\beta }}_{{{\text{cancer}}}} ) + \lambda_{{{\text{other}}}} \exp ({\mathbf{X\beta }}_{{{\text{other}}}} )}}$, $\frac{{\lambda_{{{\text{cancer}}}} \exp ({\mathbf{X\beta }}_{{{\text{cancer}}}} )}}{{\lambda_{{{\text{cardiovascular}}}} \exp ({\mathbf{X\beta }}_{{{\text{cardiovascular}}}} ) + \lambda_{{{\text{cancer}}}} \exp ({\mathbf{X\beta }}_{{{\text{cancer}}}} ) + \lambda_{{{\text{other}}}} \exp ({\mathbf{X\beta }}_{{{\text{other}}}} )}}$, and, $\frac{{\lambda_{{{\text{other}}}} \exp ({\mathbf{X\beta }}_{{{\text{other}}}} )}}{{\lambda_{{{\text{cardiovascular}}}} \exp ({\mathbf{X\beta }}_{{{\text{cardiovascular}}}} ) + \lambda_{{{\text{cancer}}}} \exp ({\mathbf{X\beta }}_{{{\text{cancer}}}} ) + \lambda_{{{\text{other}}}} \exp ({\mathbf{X\beta }}_{{{\text{other}}}} )}}$, respectively (Beyersmann et al. 2009). For each subject we generated a censoring time from an exponential distribution. The survival time was determined as the minimum of the event time and the censoring time. The rate parameter for the censoring distribution was chosen using a bisection procedure so that 25% of subjects were censored (Austin 2023).

We then fit the three cause-specific hazard models in the simulated super-population by regressing the time-to-event outcome on the 10 simulated predictor variables. The three sets of estimated regression coefficients will be considered the ‘true’ values of the regression coefficients to which the coefficients estimated below will be compared.

We then induced missing data in the simulated super-population such that the number of patterns of missing data was equal to that observed in the empirical AMI data described above (67 missing data patterns) and such that the relative frequency of the 67 patterns of missing data reflected what was observed in the AMI data above. Furthermore, we assumed a missing at random (MAR) missing data mechanism, so that, for a given predictor variable, the likelihood of missing data was related to the other predictor variables, but not to the variable itself. The missing data model for each of 8 predictor variables that were subject to missingness consisted of the 9 other predictor variables, with the regression coefficients (or weights) for these 9 variables being equal to one another. Importantly, missingness was not related to the outcome variable in the analysis model that is of scientific interest. We set data to missing such that the proportion of records that have missing data (i.e., that were incomplete) in the super-population was equal to the desired prevalence (${\text{p}}_{{{\text{missing}}}}$).

### Monte Carlo simulations: statistical analyses

The estimands of interest in the current study were the 10 coefficients in the cardiovascular death cause-specific hazard model. Of secondary interest were the 10 coefficients in the cancer death cause-specific hazard model and the 10 coefficients in the other (non-cardiovascular and non-cancer) death cause-specific hazard model.

We drew a random sample of size 1,000 without replacement from the super-population. We considered three strategies for imputation. The first strategy used the MICE algorithm to impute missing data in the random sample, with predictive mean matching (PMM) being used to impute missing variables (recent research has shown that PMM performs as well as logistic regression for imputing missing binary variables (Austin and van Buuren 2023)). The imputation model for each variable that was subject to missing data used the: (i) other nine predictor variables; (ii) the three cause-specific cumulative hazard functions and the three indicator variables denoting the event type; (iii) the interactions between the three cause-specific cumulative hazard functions and the other nine predictor variables. This strategy was based on a modification of a strategy examined by Bonneville and colleagues (Bonneville et al. 2022). The modification was that we used PMM for imputing missing continuous variables, while they used linear regression for imputing missing continuous variables.

The second strategy used the MICE algorithm to impute missing data in the random sample. The imputation model for each variable that was subject to missing data used the: (i) other nine predictor variables; (ii) the cause-specific cumulative hazard function for cardiovascular mortality and the indicator variable denoting the occurrence of a cardiovascular event; (iii) the interactions between the cause-specific cumulative hazard function for cardiovascular death and the other nine predictor variables. The rationale for including only the cause-specific cumulative hazard function for cardiovascular mortality and not the other two cause-specific cumulative hazard functions is that the primary analysis model of interest is the cause-specific hazard model for cardiovascular mortality.

The third strategy used the substantive model compatible version of fully conditional specification (SMFCS) algorithm for imputing missing data. SMCFCS uses an imputation model that is compatible (in the sense that two conditional models are compatible if there exists a joint distribution that has as conditional distributions the two conditional distributions in question (Du et al. 2022)) with all three cause-specific hazard models (in particular, it is compatible with the cardiovascular death cause-specific hazard model that is the analysis model of primary interest) (Bartlett et al. 2015). In the current study, when using SMCFCS, linear regression was used for imputing missing continuous variables while logistic regression was used for imputing missing binary variables. Auxiliary variables are variables that are not included in the analysis model but that are related to missingness. When using MICE, auxiliary variables can be included in the imputation models to improve the quality of the imputations. SMCFCS also allows for incorporating auxiliary variables. Auxiliary variables can be included in the analysis model when the compatible imputation models are derived. The auxiliary variables can then be excluded from the analysis models when these models are fit to the imputed datasets (Bartlett et al. 2015).

For all three strategies, 10 complete datasets were created (this was a pragmatic choice as using more than this number would have made the simulations too computationally burdensome, as SMCFCS imposes a substantial computational burden). In each of the complete datasets we fit the three analysis models in which a cause-specific hazard model was used to regress the cause-specific hazard function on the 10 predictor variables. The estimated regression coefficients and their standard errors were pooled across the complete datasets using Rubin’s Rules. Ninety-five percent confidence intervals were computed for each of the 10 estimated regression coefficients using normal-theory methods. Confidence intervals were constructed using Barnard and Rubin's small-sample degrees of freedom (Barnard and Rubin 1999). This process was repeated 1000 times.

For comparative purposes, we conducted a complete case analysis. Using this approach, in each random sample from the super-population, we excluded those subjects with any missing data and fit the three cause-specific hazard models using those subjects with complete data.

Finally, we repeated the entire process without setting any data to missing in the super-population. Thus, we created the super-population, but did not induce any missing data. We drew random samples of size 1,000 and fit the three cause-specific hazard models. We estimated the regression coefficients and their standard errors in each of the random samples and repeated this process 1,000 times. We refer to these scenarios as the scenarios with 0% missing data. The purpose of this set of simulations was to compare the performance of the different imputation strategies to the setting without missing data.

### Performance assessment

We primary evaluation of the performance of the three strategies for incorporating the outcome variable into the imputation model focused on two metrics: (i) the relative bias of the estimated regression coefficient for each of the 10 predictor variables; (ii) the empirical coverage rates of 95% confidence intervals. Relative bias was computed by comparing the mean estimated regression coefficient across the 1,000 simulation replicates to the corresponding regression coefficients when the analysis model was fit to the super-population. We focused on relative bias rather than absolute bias due to the variation in the magnitude of the true regression coefficients. The use of relative bias facilitated comparisons of results across the different regression coefficients. To complement the reporting of relative bias, we also report absolute bias. Empirical coverage rates of 95% confidence intervals were computed as the proportion of estimated 95% confidence that contained the true value of the regression parameter. Due to our use of 1,000 simulation replicates, an empirical coverage rate that was greater than 96.35% or less than 93.65% would be statistically significantly different from the advertised rate of 95% based on a standard normal-theory test. A secondary evaluation of the different methods compared the mean estimated standard error of each of the regression coefficients across the 1,000 simulation replicates.

### Statistical software

The simulations were conducted using the R statistical programming language (version 3.6.3). Missing data were induced using the ampute function from the mice package (version 3.13.0), while MI using the MICE algorithm was implemented using the mice function from the same package. MI using the SMCFCS algorithm was implemented using the smcfcs function from the smcfcs package (version 1.7.1). In the analyses of the samples with no missing data, the coxph function from the survival package (version 3.2–11) was used to fit the cause-specific hazard models.

## Results of Monte Carlo simulations

### Relative *bias*

**Fig. 1:**
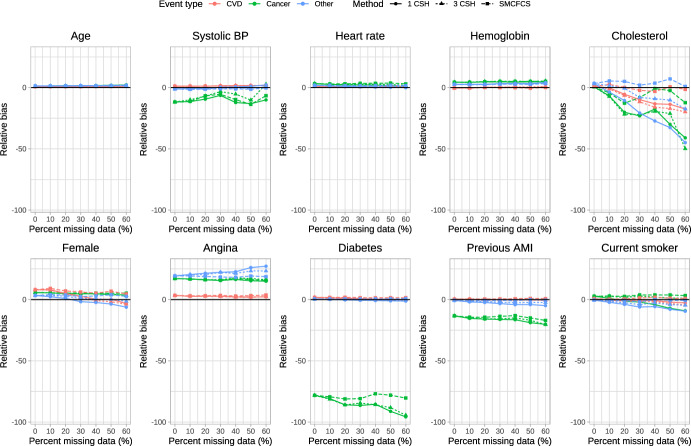
Relative bias (%) (λ_cvd_ = 1 & λ_cancer_ = 1/3 & λ_other_ = 1/3)

**Fig. 2:**
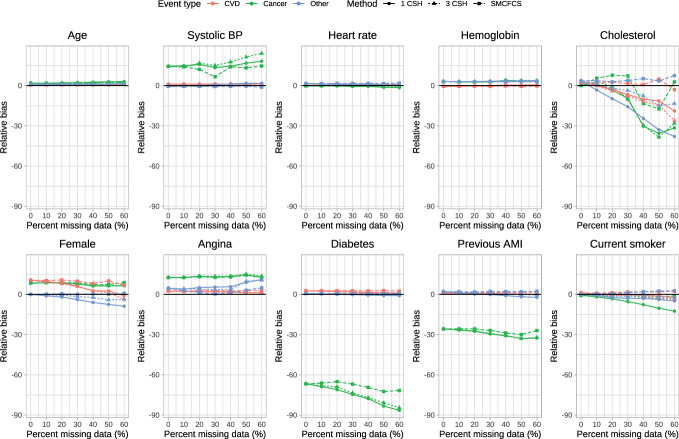
Relative bias (%) (λ_cvd_ = 1 & λ_cancer_ = 1/2 & λ_other_ = 1/2)

**Fig. 3:**
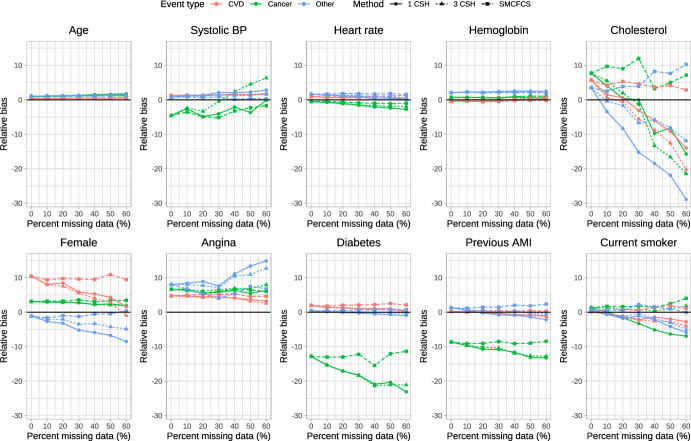
Relative bias (%) (λ_cvd_ = 1 & λ_cancer_ = 1 & λ_other_ = 1)

**Fig. 4:**
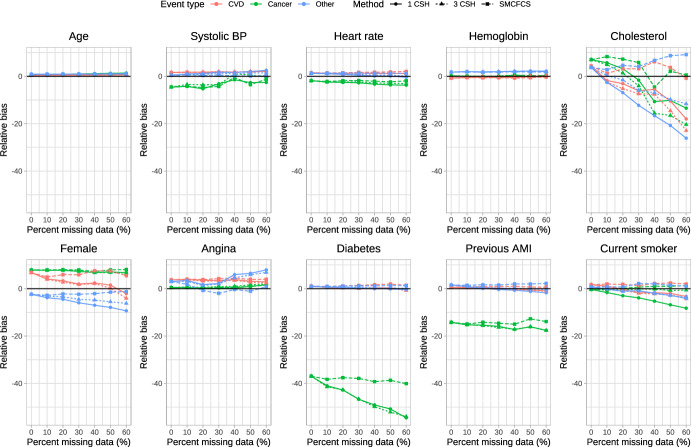
Relative bias (%) (λ_cvd_ = 1 & λ_cancer_ = 2 & λ_other_ = 2)

**Fig. 5:**
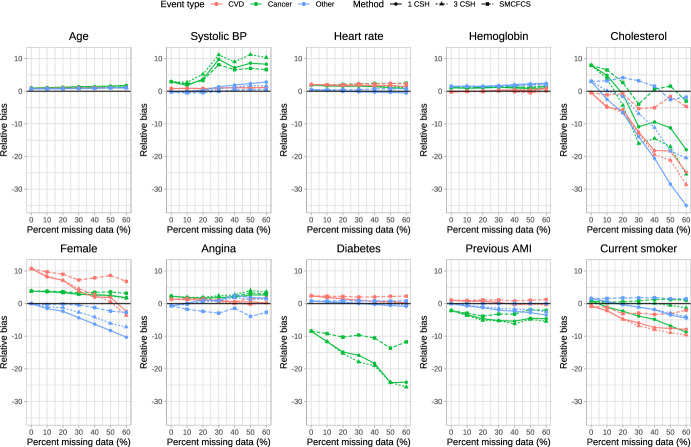
Relative bias (%) (λ_cvd_ = 1 & λ_cancer_ = 3 & λ_other_ = 3)

The relative bias of the estimated regression coefficients is reported in Fig. 1, 2, 3, 4 and 5. Due to the large relative biases observed for some of the predictor variables in the complete case analysis, we do not report the relative bias for the complete case analysis in these figures. The relative bias for the complete case analysis is reported in Figures A1 to A5 in the supplemental online material. There is one figure for each of the sets of rate parameters ($\lambda_{{{\text{cardiovascular}}}}$, $\lambda_{{{\text{cancer}}}}$, and $\lambda_{{{\text{other}}}}$). Each figure consists of 10 panels, one for each of the predictor variables in the cause-specific hazard models (e.g., the top-left panel in each figure reports relative bias in estimating the regression coefficient for age). Each panel contains nine lines describing the relationship between relative bias and the prevalence of missing data for the different combinations of the three cause-specific hazard models and the three imputation strategies. Within a given panel, the same colour is used for reporting the results for estimating the same cause-specific hazard model (e.g., red is used for reporting the relative bias in estimating the coefficients of the cause-specific hazard model for cardiovascular death). The same scale is used for the vertical axis in all 10 panels, to allow the relative bias to be compared across the 10 variables. Within a given panel, the important comparison is between the different lines of the same colour (i.e., comparing the different strategies for imputation when estimating the coefficients of the same cause-specific hazard function). Note that to each panel we have added the results for the cause-specific analyses when there were no missing data (labeled as having a 0% prevalence of missing data). To complement the reporting of relative bias, we report bias in Figures A6 through A10 in the supplemental online material.

When $\lambda_{{{\text{cardiovascular}}}} = 1,\;\lambda_{{{\text{cancer}}}} = \frac{1}{3},\;\lambda_{{{\text{other}}}} = \frac{1}{3}$ (Fig. 1), there were some predictor variables for which there was very little variation in relative bias between the three imputation strategies across all three cause-specific hazard models (e.g., age, heart rate, and hemoglobin), regardless of the proportion of missing data. For other variables, there were little differences in relative bias for the three imputation strategies for some of the cause-specific hazard models, but differences in relative bias between the three imputation strategies for other cause-specific hazard models (e.g., systolic blood pressure and diabetes). For systolic blood pressure, the use the three cause-specific hazard functions resulted in estimates of the log-hazard ratio for the cancer mortality hazard model (green lines) with little bias when the prevalence of missing data was high, whereas the use of the other two imputation strategies resulted in estimates of the log-hazard ratio with modest bias when the prevalence of missing data was high. For cholesterol in each of the three cause-specific hazard models, the use of SMCFCS tended to result in modestly less bias compared to the use of the two other imputation strategies regardless of the prevalence of missing data. For the cancer cause-specific model (green line), the relative bias in estimating the coefficient for diabetes was lower when using SMCFCS compared to when the other two imputation strategies were used (note that the moderately large relative bias that we observed when there were no missing data is likely due to the use of relative bias coupled with a small true log-hazard ratio; see supplemental results on absolute bias). Similar results were observed in the other four scenarios defined by different combinations of rate parameters.

The primary comparison of interest is for estimating of the cardiovascular cause-specific hazard model (red lines in each panel). In general, SMCFCS tended to result in estimates with lower relative bias. However, this was not universally true. For instance, when $\lambda_{{{\text{cardiovascular}}}} = 1,\;\lambda_{{{\text{cancer}}}} = 1,\;\lambda_{{{\text{other}}}} = 1$ (Fig. 3), SMCFCS resulted in greater relative bias than did the other two imputation strategies when estimating the female sex coefficient. Similarly, when $\lambda_{{{\text{cardiovascular}}}} = 1,\;\lambda_{{{\text{cancer}}}} = 2,\;\lambda_{{{\text{other}}}} = 2$ (Fig. 4), SMCFCS tended to result in greater relative bias than did the other two imputation strategies when estimating the female sex coefficient.

The relative bias in the complete case analysis are reported in Figures A1 to A5 in the online supplemental material. For some predictor variables in some scenarios, the relative bias in the cancer cause-specific hazard model were very large when the prevalence of missing data was high.

In synthesizing these results, we focus on two coefficients in the cardiovascular death cause-specific hazard model: cholesterol (which was the variable with the greatest amount of missingness) and female sex. For cholesterol, the use of SMCFCS tended to result in estimates with lower relative bias than did the two MICE-based strategies, particularly when the prevalence of missing data was moderate to high. For female sex, the use of SMCFCS tended to result in estimates with greater relative bias than did the two MICE-based strategies when the prevalence of missing data was moderate to high. However, the relative bias with SMCFCS for female sex tended to be lower than the relative bias of the MICE-based strategies for cholesterol. This suggests, that, in general, SMCFCS may be a preferable strategy.

### Coverage of 95% confidence intervals

**Fig. 6:**
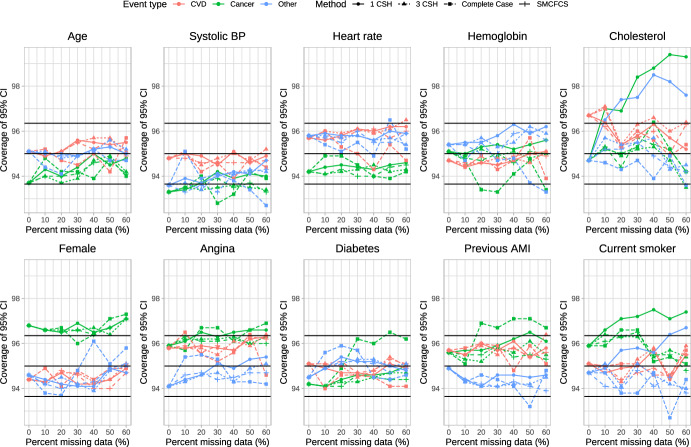
Coverage of 95% CI (%) (λ_cvd_ = 1 & λ_cancer_ = 1/3 & λ_other_ = 1/3)

**Fig. 7:**
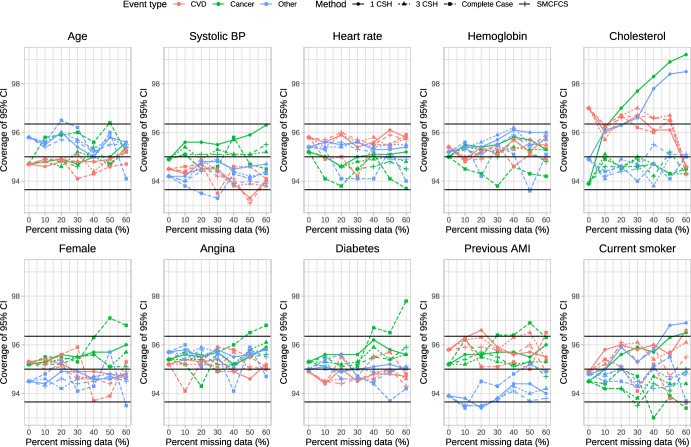
Coverage of 95% CI (%) (λ_cvd_ = 1 & λ_cancer_ = 1/2 & λ_other_ = 1/2)

**Fig. 8:**
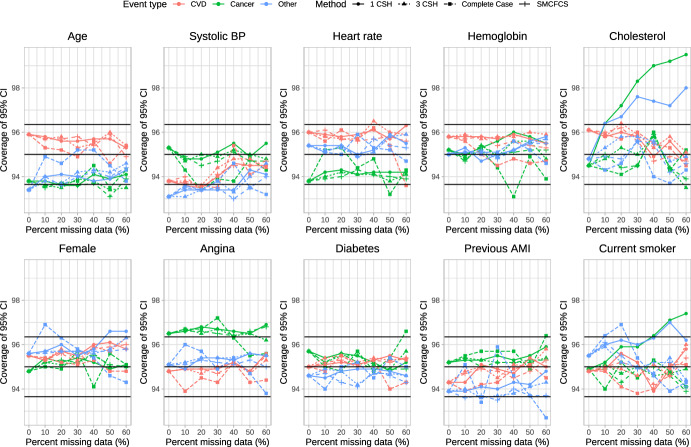
Coverage of 95% CI (%) (λ_cvd_ = 1 & λ_cancer_ = 1 & λ_other_ = 1)

**Fig. 9:**
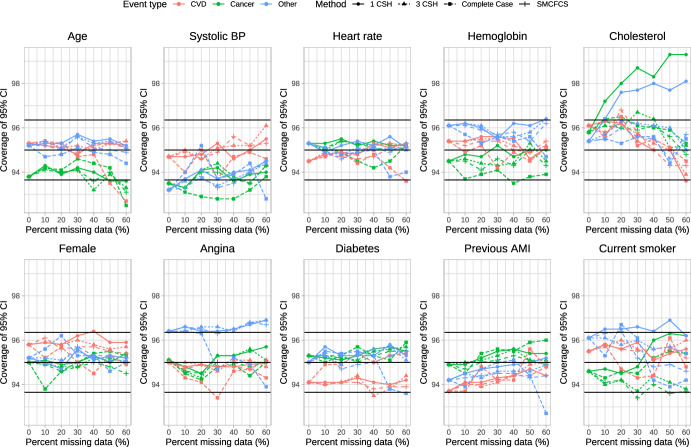
Coverage of 95% CI (%) (λ_cvd_ = 1 & λ_cancer_ = 2 & λ_other_ = 2)

**Fig. 10:**
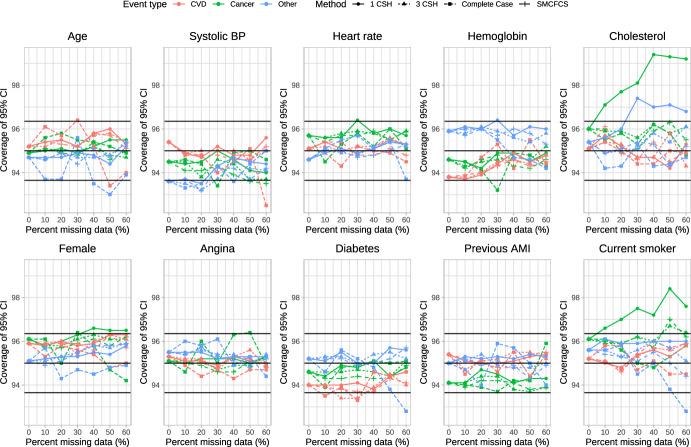
Coverage of 95% CI (%) (λ_cvd_ = 1 & λ_cancer_ = 3 & λ_other_ = 3)

The empirical coverage rates of estimated 95% confidence intervals is reported in Figs. 6, 7, 8, 9 and 10. These figures have a similar structure to Figs. 1, 2, 3, 4 and 5. On each panel we have superimposed three horizontal lines. The middle horizontal line denotes the advertised coverage rate of 95%. The lower and upper horizontal lines denote empirical coverage rates of 93.65% and 96.35%, respectively. As noted above, empirical coverage rates that lie below 93.65% or above 96.35% are statistically significantly different from the advertised rate of 95% using a standard normal-theory test. In most scenarios and for most variables, the three imputation strategies resulted in 95% confidence intervals that either had the advertised coverage rates (i.e., empirical coverage rates that lay between 93.65% and 96.35%) or that were conservative (i.e., empirical coverage rates that exceeded 95%).

Frequently, the complete case analysis resulted in estimated 95% confidence intervals whose empirical coverage rates did not differ from the advertised rate. However, there were a few scenarios and a few variables for which the empirical coverage rates were slightly lower than advertised.

### Estimated standard errors

The mean estimated standard errors are reported in Figures A11 through A15 in the supplemental online material. There are two primary observations that merit highlighting. First, for the complete case analysis, the estimated standard error tended to increase as the prevalence of missing data increased. Second, for most of the predictor variables, the prevalence of missing data had at most a marginal effect on the mean estimated standard error for each of the three imputation methods. The primary exception to this was for cholesterol, the variable for which there was the most missing data. For cholesterol, the mean estimated standard error tended to increase with an increasing prevalence of missing data, although the effect was attenuated compared to what was observed for the complete case analysis.

## Case study

We provide a case study to examine differences between the three imputation strategies in a specific set of empirical analyses. We used the data described in Sect. 2.1 consisting of 19,395 patients hospitalized with AMI. The analysis models of scientific interest were the three cause-specific hazard models described above. Using each of the three strategies for imputations, we created 48 complete datasets (White et al. 2011). In each of the 48 imputed datasets we fit the three cause-specific hazard models and pooled the estimated regression coefficients and their associated standard errors using Rubin’s Rules. We also conducted a complete case analysis in which we excluded all subjects with any missing data on any of the predictor variables.

**Fig. 11:**
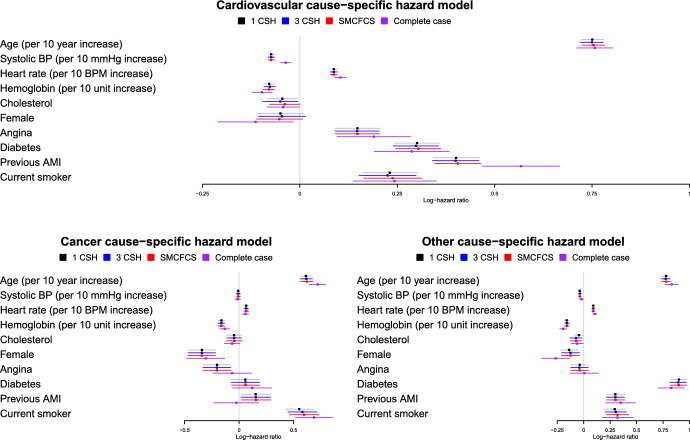
Estimated log − hazard ratios and 95% confidence intervals

The estimated log-hazard ratios and their associated 95% confidence intervals are reported in Fig. 11. The figure consists of three panels, one for each of the cause-specific hazard models. For a given predictor variable and type of outcome, the estimated log-hazard ratios and their associated 95% confidence intervals were very similar across the three imputation strategies. In a few instances (e.g., history of previous AMI in the cardiovascular cause-specific model), the estimated log-hazard ratio for the complete case analysis was substantially different from those obtained using the three imputation strategies. Furthermore, the estimated 95% confidence intervals for the complete case analysis were often wider than those obtained using multiple imputation, and at times substantially wider.

## Discussion

We compared the relative performance of two MICE-based imputation strategies with that of SMCFCS using a simulation whose design reflected a clinically realistic setting. We found that no imputation strategy consistently resulted in estimates with lower relative bias than the other strategies. Furthermore, all strategies tended to produce estimated 95% confidence intervals whose empirical coverage rates were either no different from the advertised rate or that were conservative. However, based on a comparison of the magnitudes of the relative biases for the different approaches across different variables and different scenarios, one can argue that, in general, SMCFCS may be the preferred strategy.

To the best of our knowledge, only two studies have examined the use of multiple imputation in settings with competing risks. Bartlett and Taylor used simulations to compare different MICE-based imputation strategies with the use of SMCFCS in a setting with two types of events (Bartlett and Taylor 2016). Their simulation design had two binary predictor variables and a single continuous predictor variable, with only the latter being subject to missing data. Fifty percent of subjects were subject to missing data. The regression coefficients for the first cause-specific hazard model were all equal to 1, while the regression coefficients for the second cause-specific hazard model were equal to 0.5, − 1, and 0.75. While they considered several different imputation strategies, we focus on two when summarizing their study (as these were the two best performing strategies in their study and both were examined in the current study). The first strategy was the use of MICE with the inclusion of the two binary predictor variables, the event type variable, the two cumulative cause-specific hazard functions, and the interactions of the two cause-specific hazard functions with two binary predictor variables being included in the imputation model for the continuous variable that was subject to missingness. The second strategy was SMCFCS with the substantive models being the two cause-specific hazard models. In general, the use of SMFCS resulted in estimates with less bias than did the MICE-based imputation strategy. Their results contrast with our results, which found that, while SMCFCS often had very good performance, neither approach was consistently superior to the other. There are several possible reasons for this divergence of results. First, their design included three predictor variables, while our included 10 predictor variables. Second, their design used stronger predictor effects than did our design. The regression coefficients in the current study were obtained from empirical analyses of patients hospitalized with AMI, thus the predictor effects in the current study may be more reflective of what is seen in clinical research. Third, while they fixed the prevalence of missing data to 0.5 (50%), we allowed it to vary from 0.1 (10%) to 0.6 (60%). Fourth, their design had one pattern of missing data (i.e., only one covariate was subject to missing data), while our design, which was based on an analysis of empirical data, had 67 patterns of missing data. Fifth, their design had two event types, while the current study had three event types. In a similar study, Bonneville et al. used simulations to compare the performance of SMCFCS with three MICE-based strategies for multiple imputation in the presence of competing risks (Bonneville et al. 2022). The MICE-based strategies differed based on which cumulative cause-specific hazard functions were included in the imputation models and whether interactions with these terms were included in the imputation model. Their simulation design had two predictor variables, with only the first covariate being subject to missingness; thus, as with Bartlett and Taylor, there was only one pattern of missing data. Similarly, as with Bartlett and Taylor, there were two types of events. With a weak covariate effect (cause-specific hazard ratio of e^0.5^ for the variable subject to missingness in the model for the first event type), differences in relative bias tended to be minor between SMCFCS and the MICE method that incorporated both cause-specific hazard functions and their interactions. With a strong covariate effect (cause-specific hazard ratio of e^1^), the MICE method displayed greater bias when the prevalence of missing data was high, whereas SMCFCS resulted in estimates with little bias. Finally, while they did not formally compare different imputation strategies, Lau and Lesko discuss issues in missing data in settings with competing risks (Lau and Lesko 2018). They highlighted the use of SMCFCS and of conventional MICE with the inclusion of the cumulative baseline hazard functions for the different event types, along with their event indicator variables, in the imputation models. They state that “there has been even less research on multiple imputation for missing covariate values in the context of time-to-event outcomes when there are competing events”. The current study was intended to address the paucity of research on this topic. The current study complements that of Bonneville et al. and Bartlett and Taylor in several ways. First, while they considered settings with two or three predictor variables, we considered a setting with 10 predictor variables. Second, while their scenarios had one pattern of missing data, our design had 67 patterns of missing data. The covariate effects in the current simulations were obtained from empirical analyses of patients hospitalized with AMI. Thus, our simulations suggest that, in a clinically-realistic setting, differences between the approaches were often minimal. Furthermore, while SMFCS often had superior performance, no method had uniformly superior performance to the other methods.

The current study is subject to certain limitations. First, our study relied on Monte Carlo simulations. Consequently, our findings are dependent on the data-generating process that was used. A strength of our simulations was the use of a data-generating process that was based on empirical analyses of patients hospitalized with AMI. Thus, we compared the performance of different strategies for imputing missing data in the presence of competing risks in a clinically realistic setting. Second, in our simulations we assumed that each of the three cause-specific hazard functions was constant over time (i.e., that cause-specific event times followed an exponential distribution). The rationale for this decision was pragmatic. One could elect to simulate cause-specific outcomes using the latent failure time approach which would allow for more complex cause-specific hazard functions (the latent failure time approach requires generating time-to-event outcomes for each of the cause-specific outcomes and then choosing the minimum of the cause-specific event times). However, the use of the latent failure time approach to simulating competing risk data has been severely criticized for both lack of plausibility in medical settings and for non-identifiability reasons (Beyersmann et al. 2009). Given the mathematical tractability of the exponential distribution, we elected to use that distribution to avoid the use of the criticized latent failure time approach. In theory one could use cause-specific hazard approach with more complex hazard functions, however this approach requires the use of numerical integration methods that do not lend themselves well to complex Monte Carlo simulations. Third, in our simulations, we created 10 complete datasets in each iteration of the simulations, regardless of the proportion of subjects who had any missing data. Both von Hippel and White and colleagues suggested that, as a rule-of-thumb, the number of imputed datasets should be equal to the percentage of subjects with any missing data (von Hippell 2009; White et al. 2011), as this will ensure reproducibility of estimated coefficients, standard errors, and p-values across replications of the entire imputation process. We would note that their recommendations are based on reproducibility of estimated quantities across replications of the imputation process and are not specific to a specific form of imputation. Due to the computational burden of using SMCFCS, we were not able to set the number of imputed datasets to equal the percentage of subjects with missing data. In order to have a fair comparison of all imputation methods, we fixed the number of imputed datasets to be equal across imputation strategies. In applied settings, analysts may elect to use a different number of imputed datasets.

In conclusion, in a simulation setting that reflected a clinically-realistic scenario, we found that neither of the two MICE-based imputation strategies and SMCFCS had consistently superior performance in terms of relative bias. All three strategies tended to produce confidence intervals with the correct coverage rates. Based on a comparison of the magnitudes of relative biases, SMCFCS may be the preferred strategy in settings that reflect our simulation design.

## Supplementary Information

Below is the link to the electronic supplementary material.

Supplementary file 1 (PDF 7247 kb)
