# Supplementary material for: Multiple imputation with competing risk outcomes
Source: Comput Stat. 2024 Jun 26;40(2):929–49. doi: 10.1007/s00180-024-01518-w (PMC11832607; doi:10.1007/s00180-024-01518-w)

Figure A 1 . Relative bias (%) for complete case analysis ($\lambda_{cvd} = 1$ & $\lambda_{cancer} = 1/3$ & $\lambda_{other} = 1/3$)

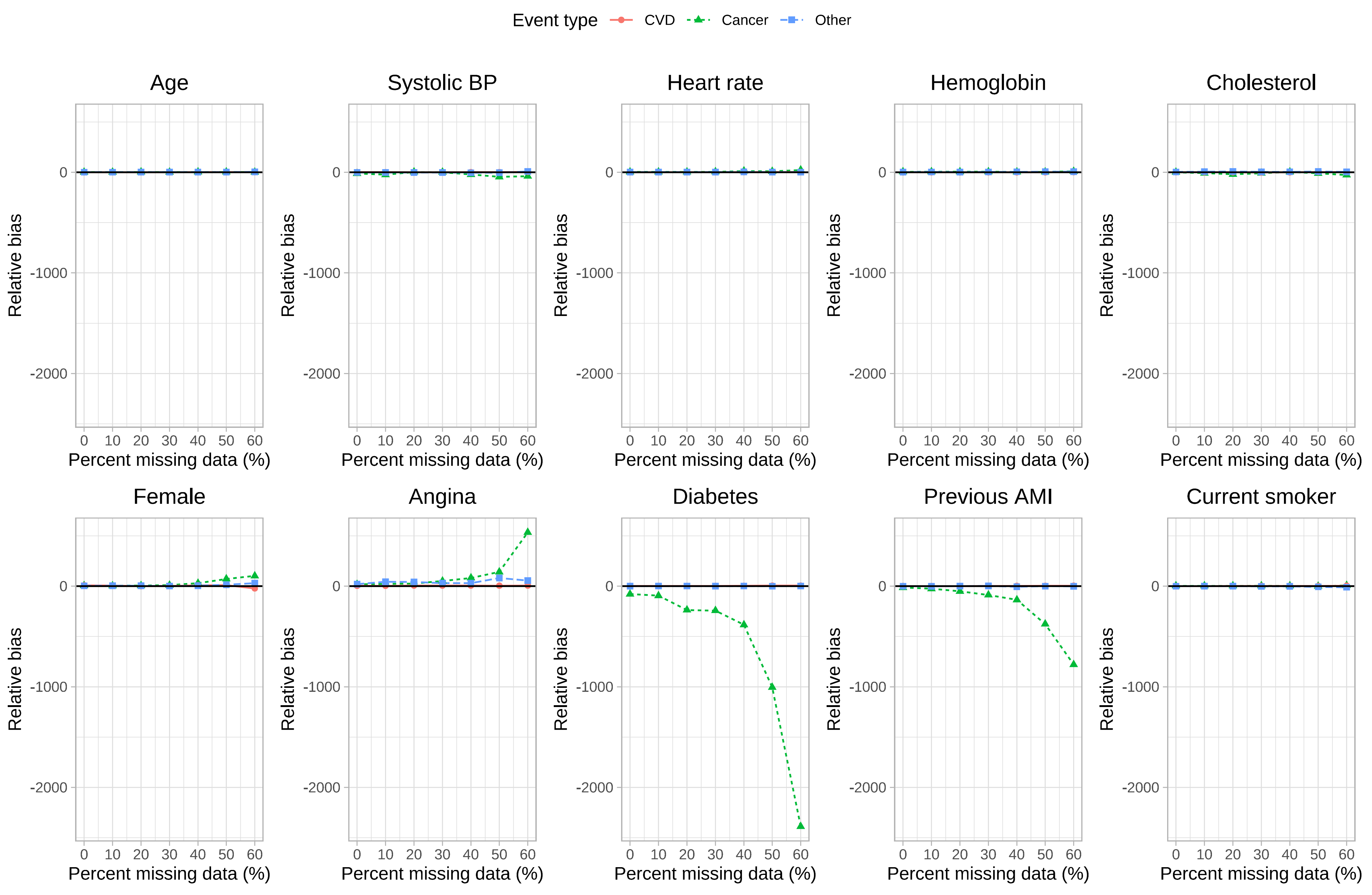

Figure A 2 . Relative bias (%) for complete case analysis (λ_{cvd} = 1 & λ_{cancer} = 1/2 & λ_{other} = 1/2)

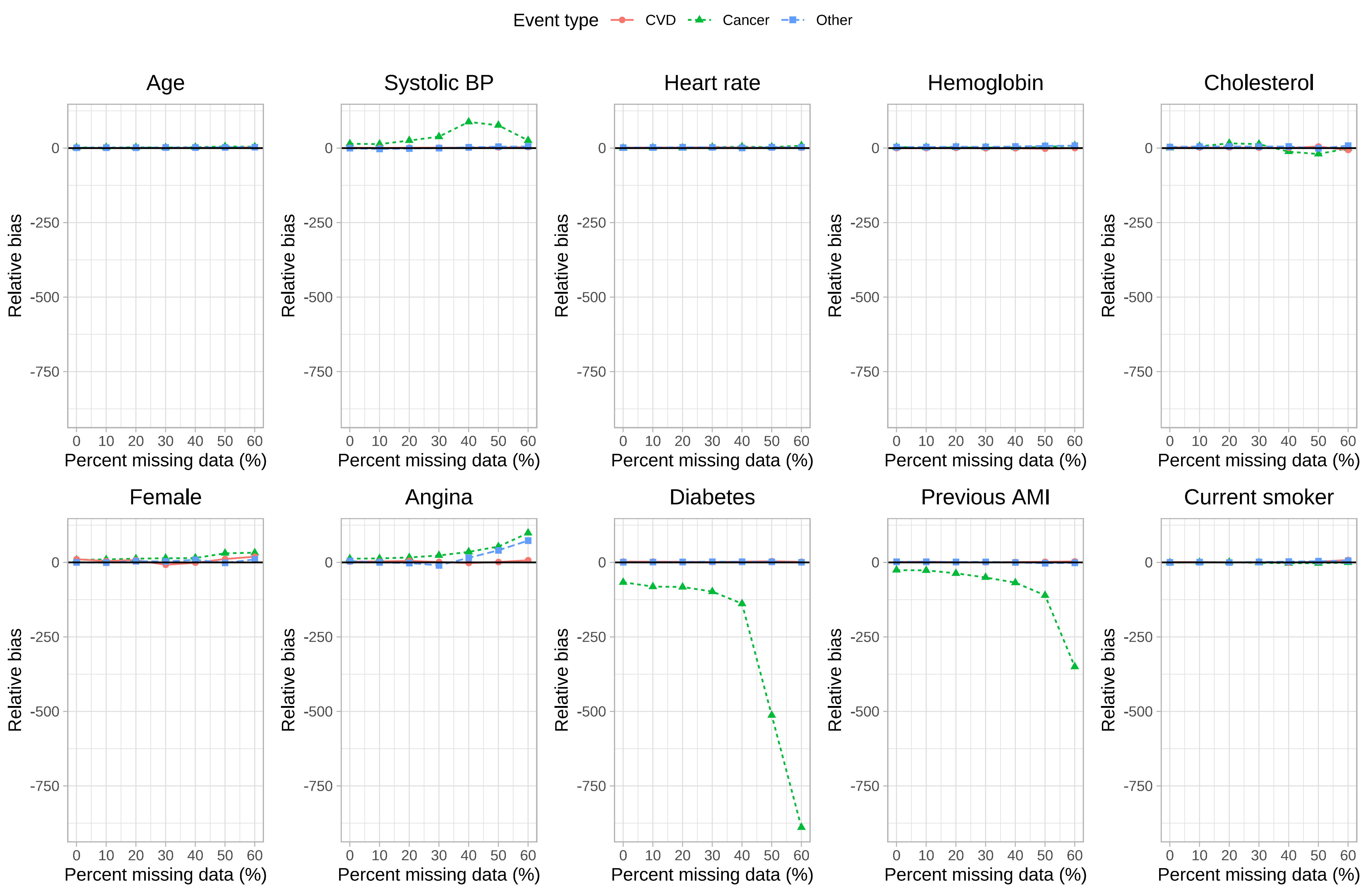

Figure A 3 . Relative bias (%) for complete case analysis ($\lambda_{cvd} = 1$ & $\lambda_{cancer} = 1$ & $\lambda_{other} = 1$)

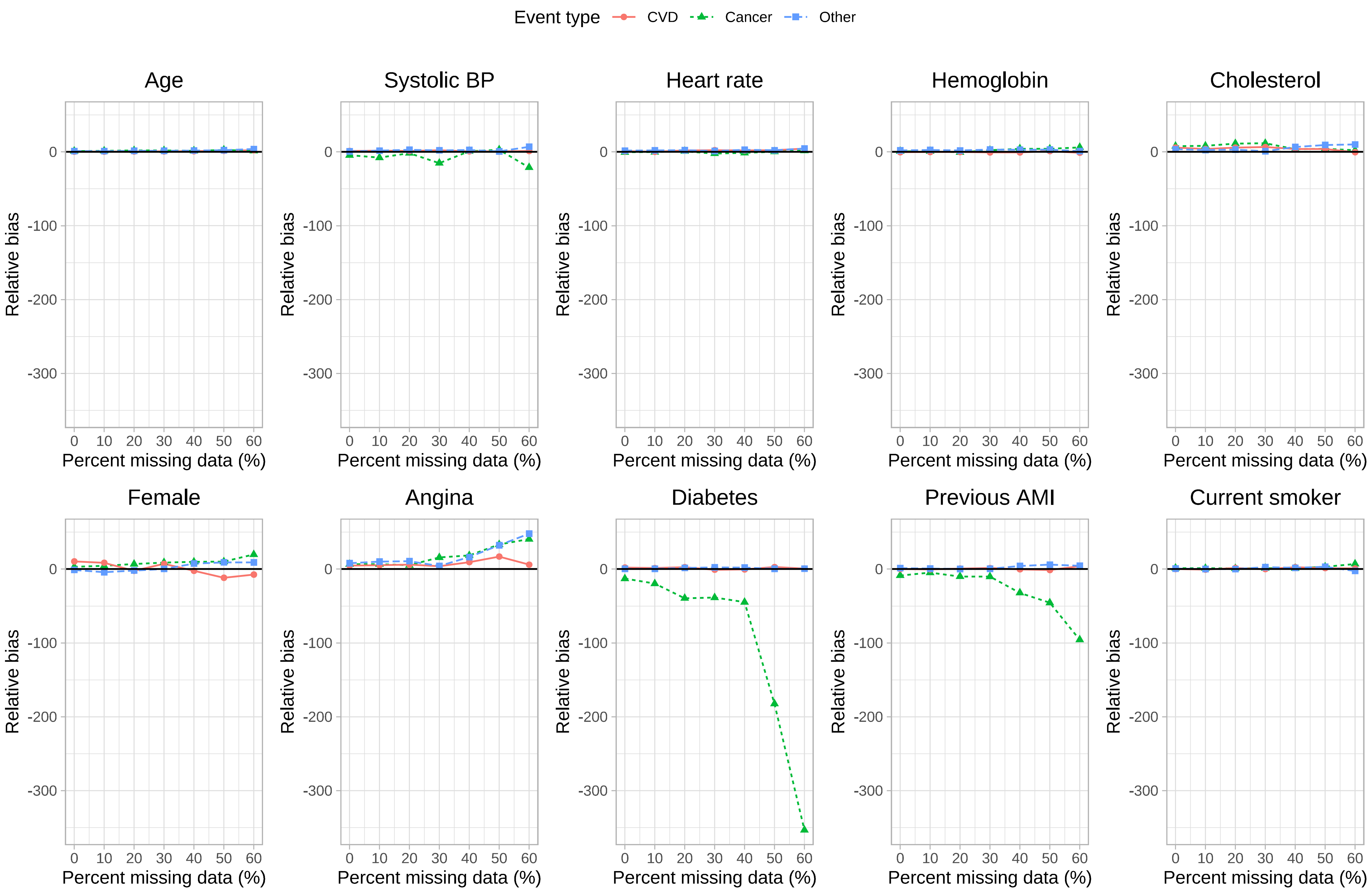

Figure A 4 . Relative bias (%) for complete case analysis ($\lambda_{cvd} = 1$ & $\lambda_{cancer} = 2$ & $\lambda_{other} = 2$)

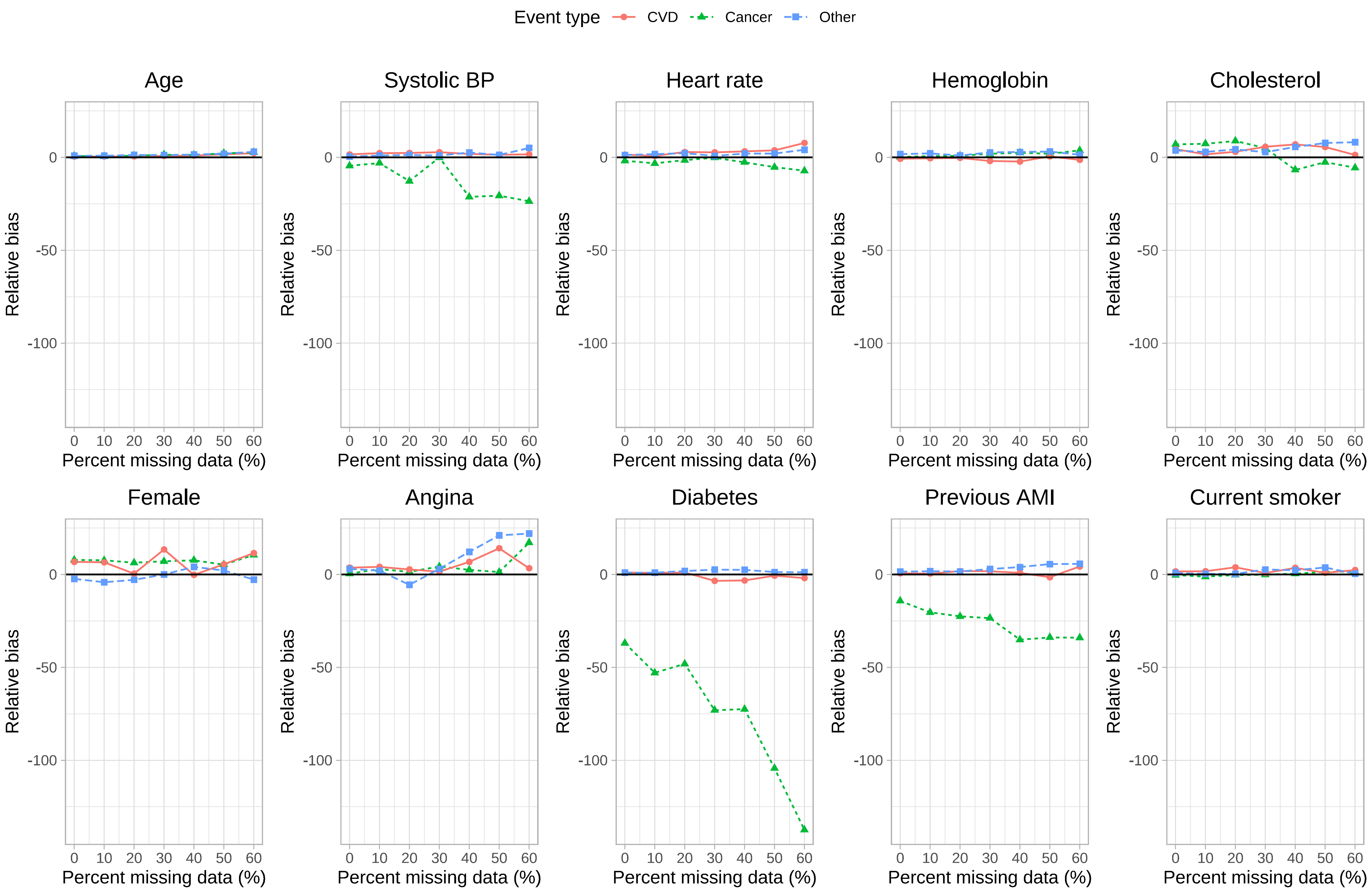

Figure A 5 . Relative bias (%) for complete case analysis ($\lambda_{cvd} = 1$ & $\lambda_{cancer} = 3$ & $\lambda_{other} = 3$)

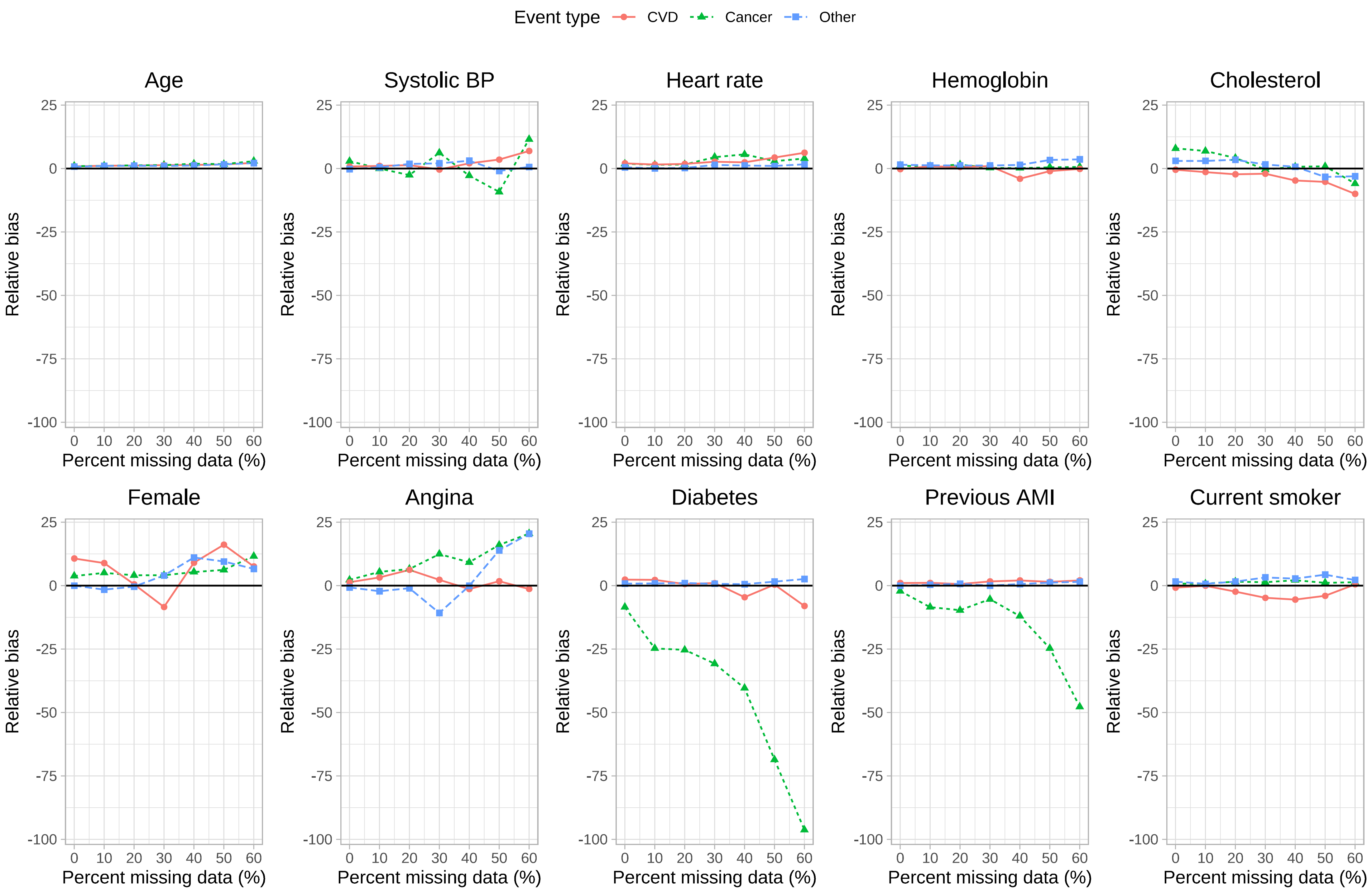

Figure A 6 . Bias (%) (λ_{cvd} = 1 & λ_{cancer} = 1/3 & λ_{other} = 1/3)

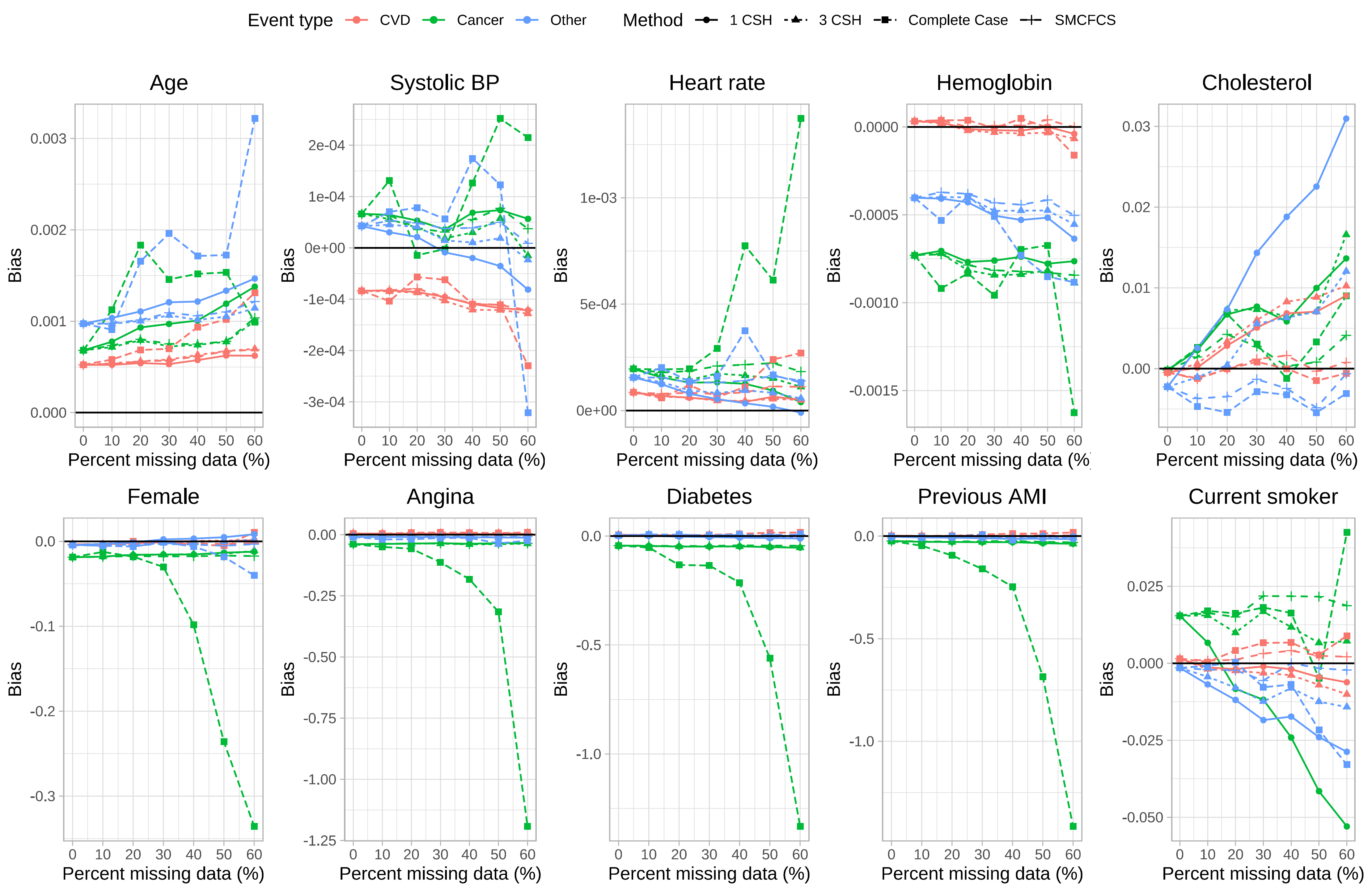

Figure A 7 . Bias (%) ($\lambda_{cvd} = 1$ & $\lambda_{cancer} = 1/2$ & $\lambda_{other} = 1/2$)

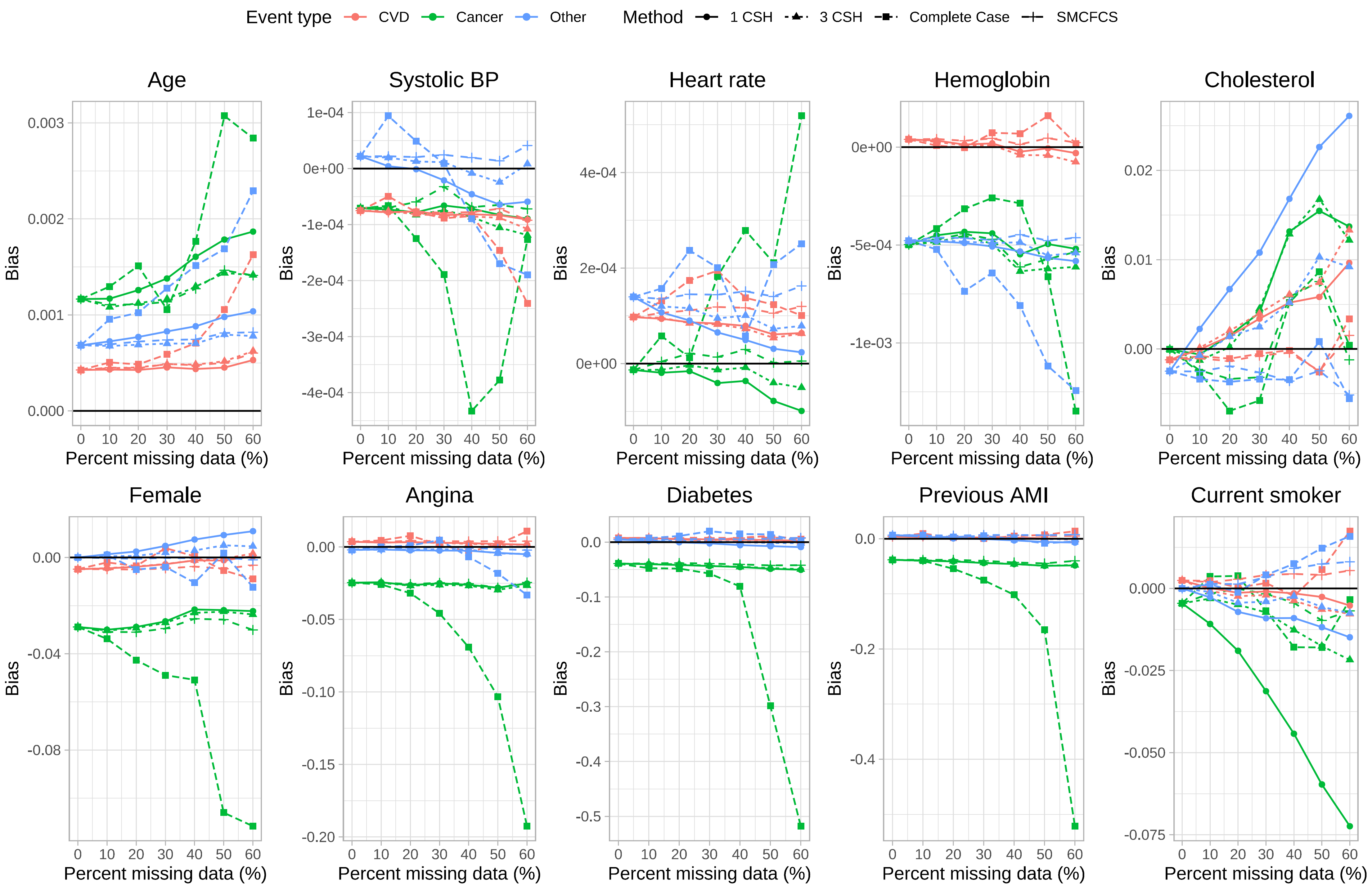

Figure A 8 . Bias (%) (λ_{cvd} = 1 & λ_{cancer} = 1 & λ_{other} = 1)

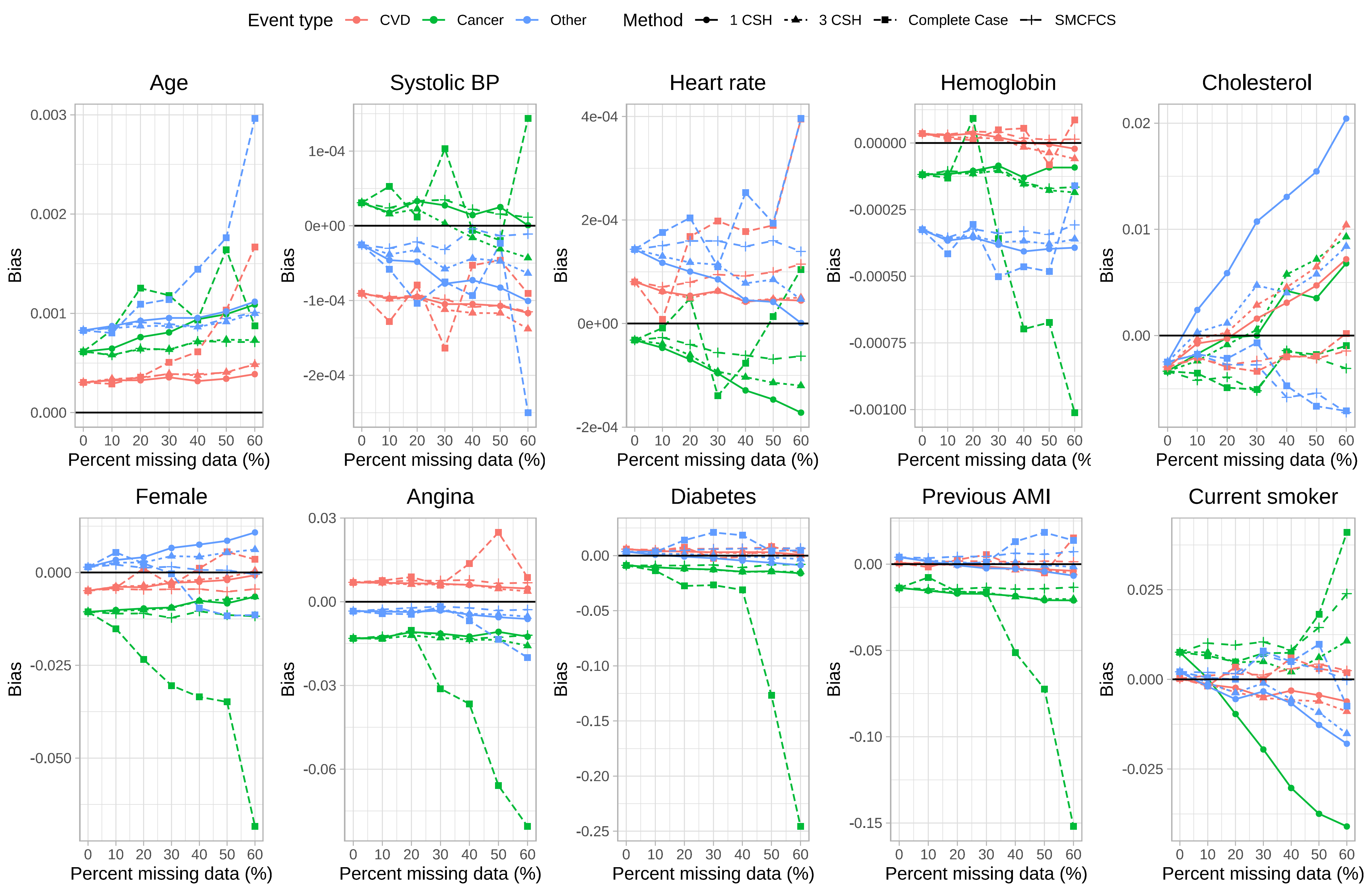

Figure A 9 . Bias (%) ($\lambda_{cvd} = 1$ & $\lambda_{cancer} = 2$ & $\lambda_{other} = 2$)

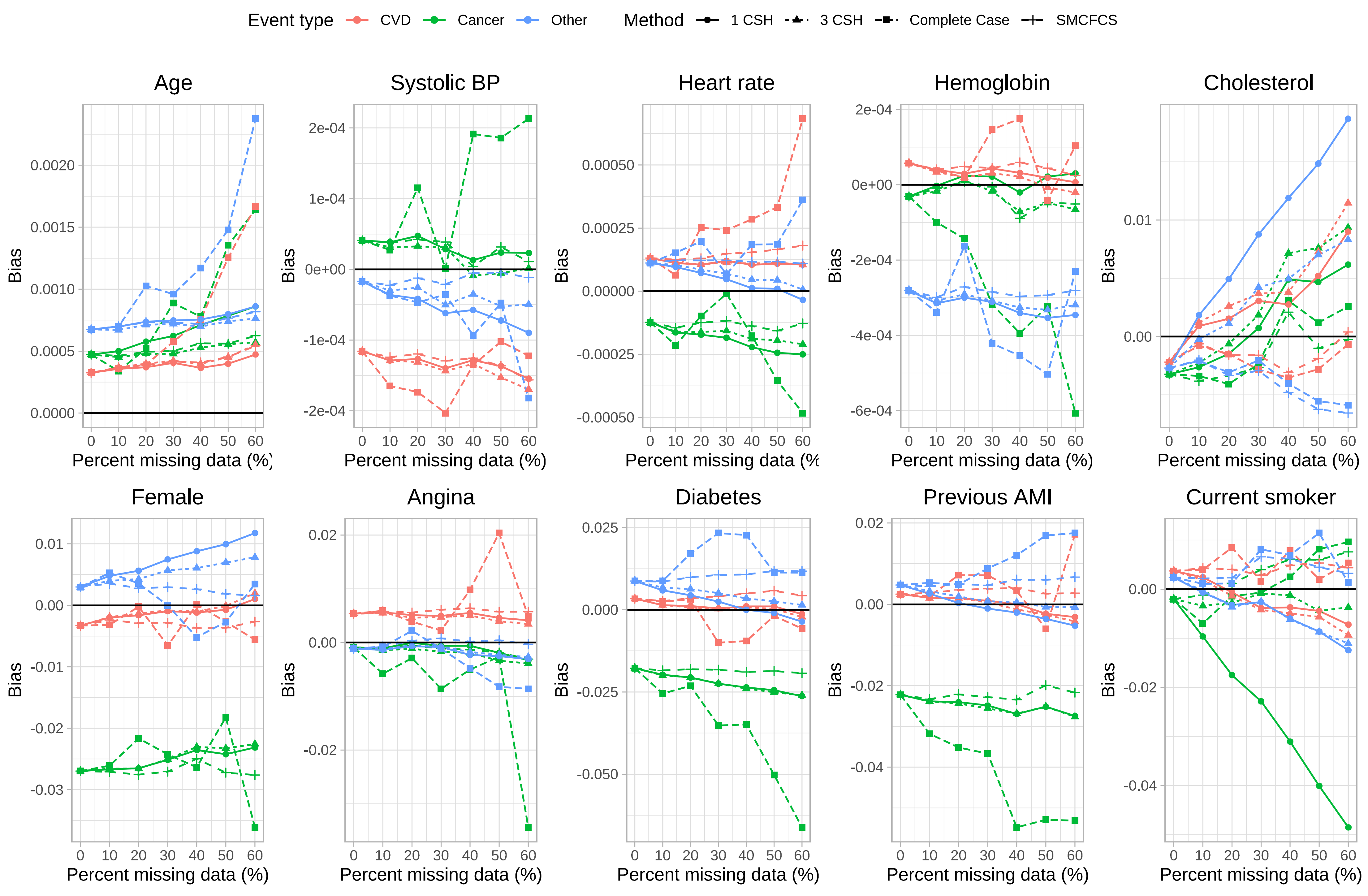

Figure A 10 . Bias (%) (λ_{cvd} = 1 & λ_{cancer} = 3 & λ_{other} = 3)

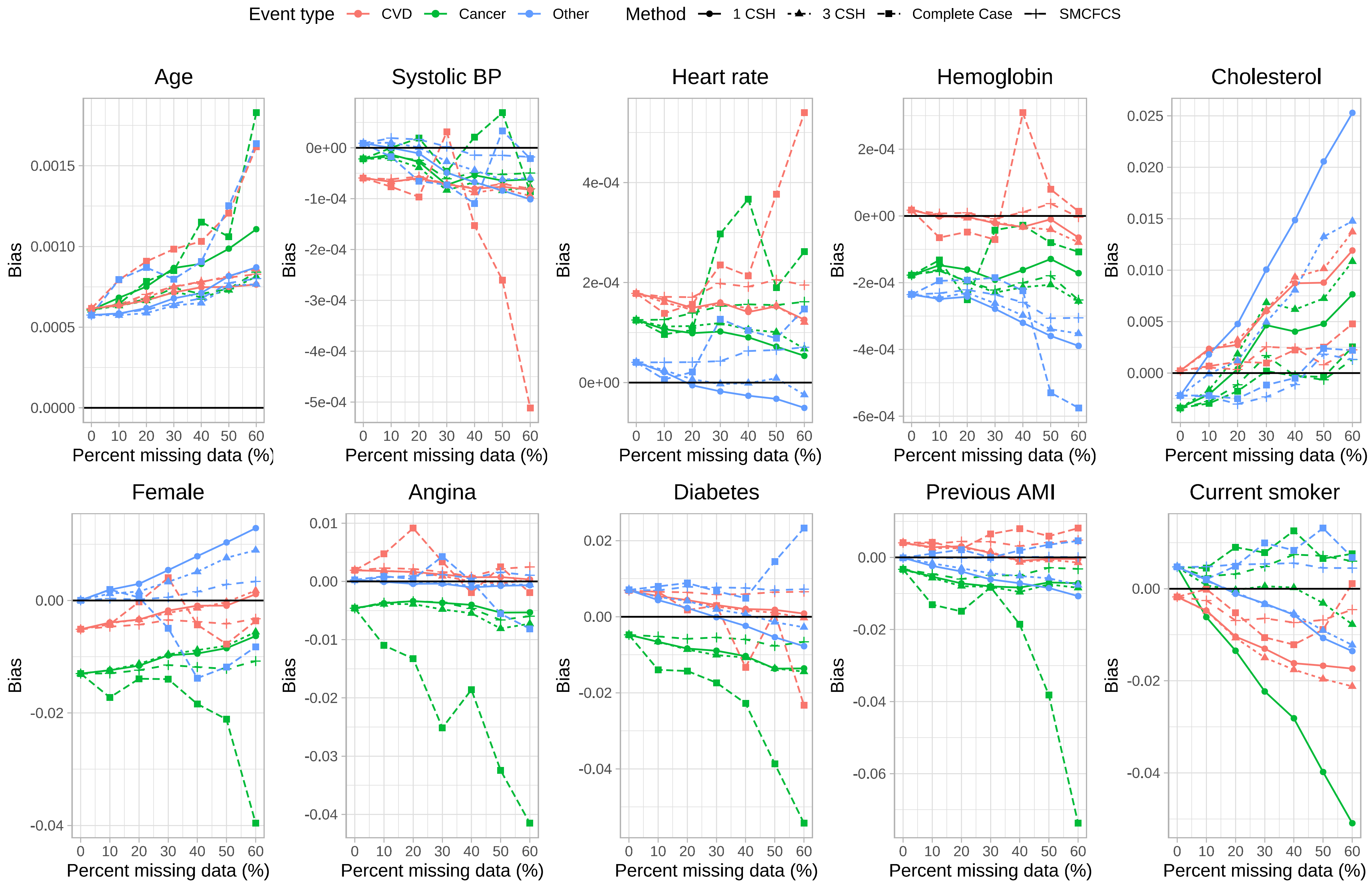

Figure A 11 . Mean standard error ($\lambda_{cvd} = 1$ & $\lambda_{cancer} = 1/3$ & $\lambda_{other} = 1/3$)

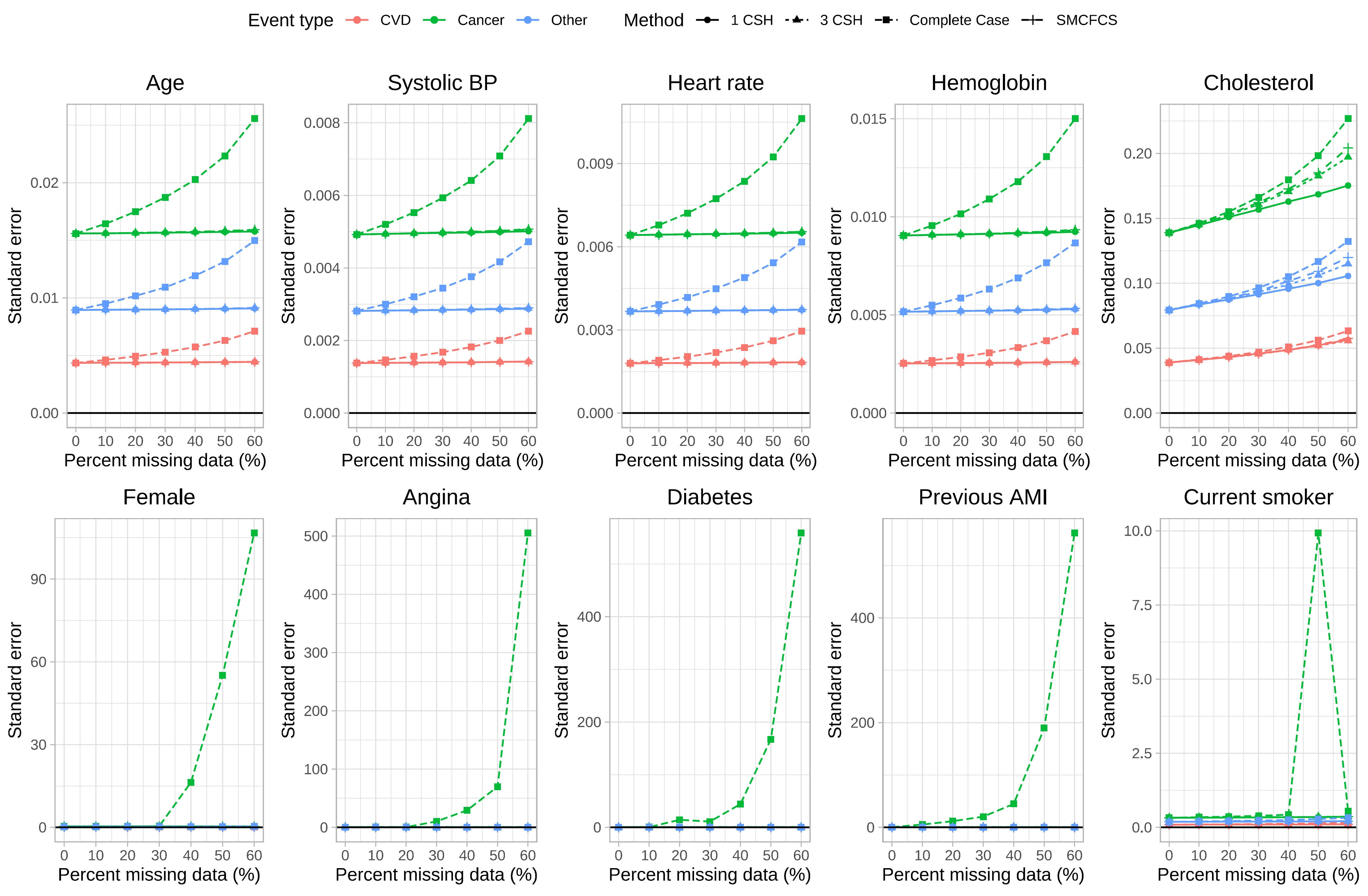

Figure A 12 . Mean standard error (λ_{cvd} = 1 & λ_{cancer} = 1/2 & λ_{other} = 1/2)

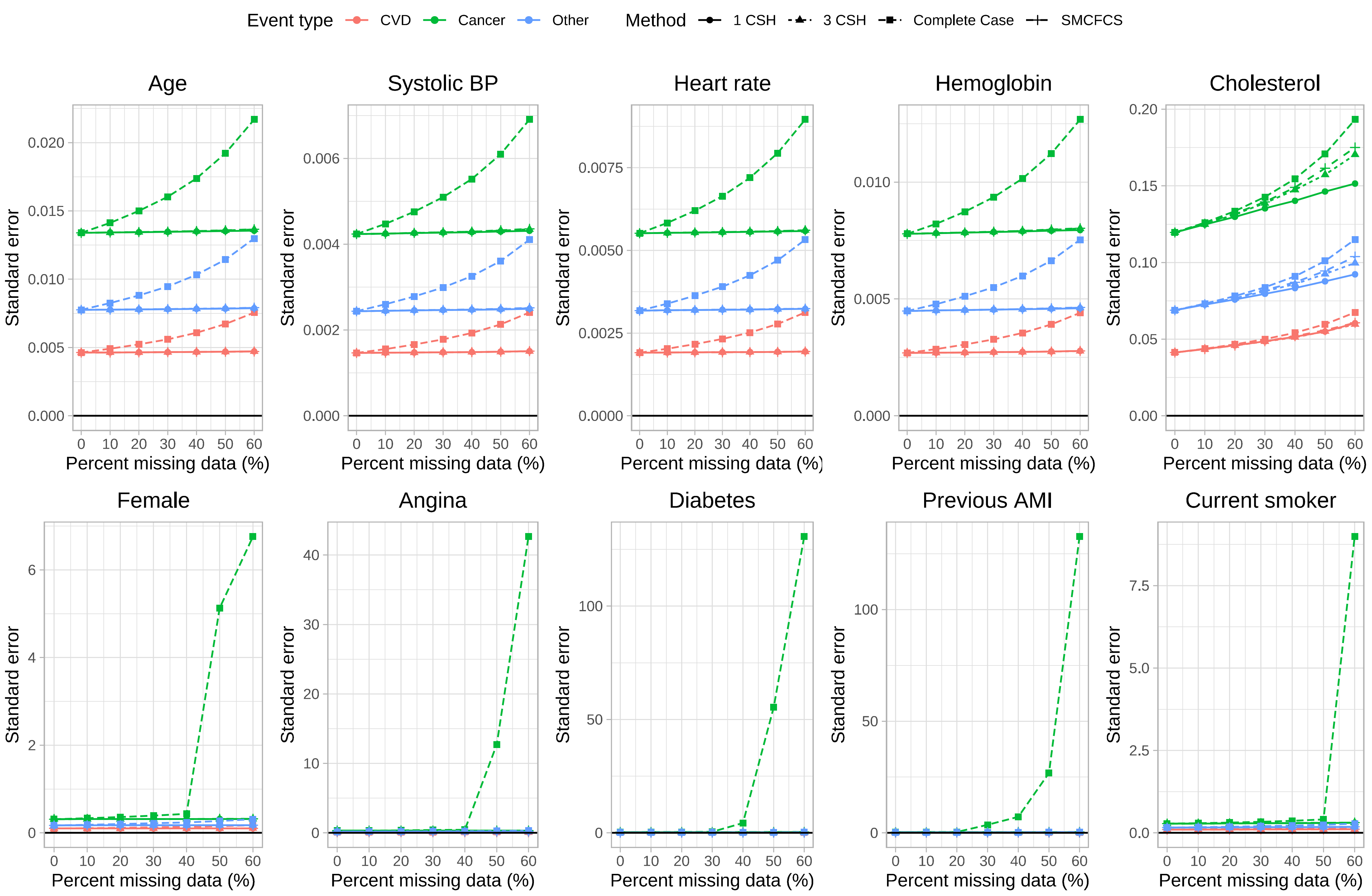

Figure A 13 . Mean standard error ($\lambda_{cvd} = 1$ & $\lambda_{cancer} = 1$ & $\lambda_{other} = 1$)

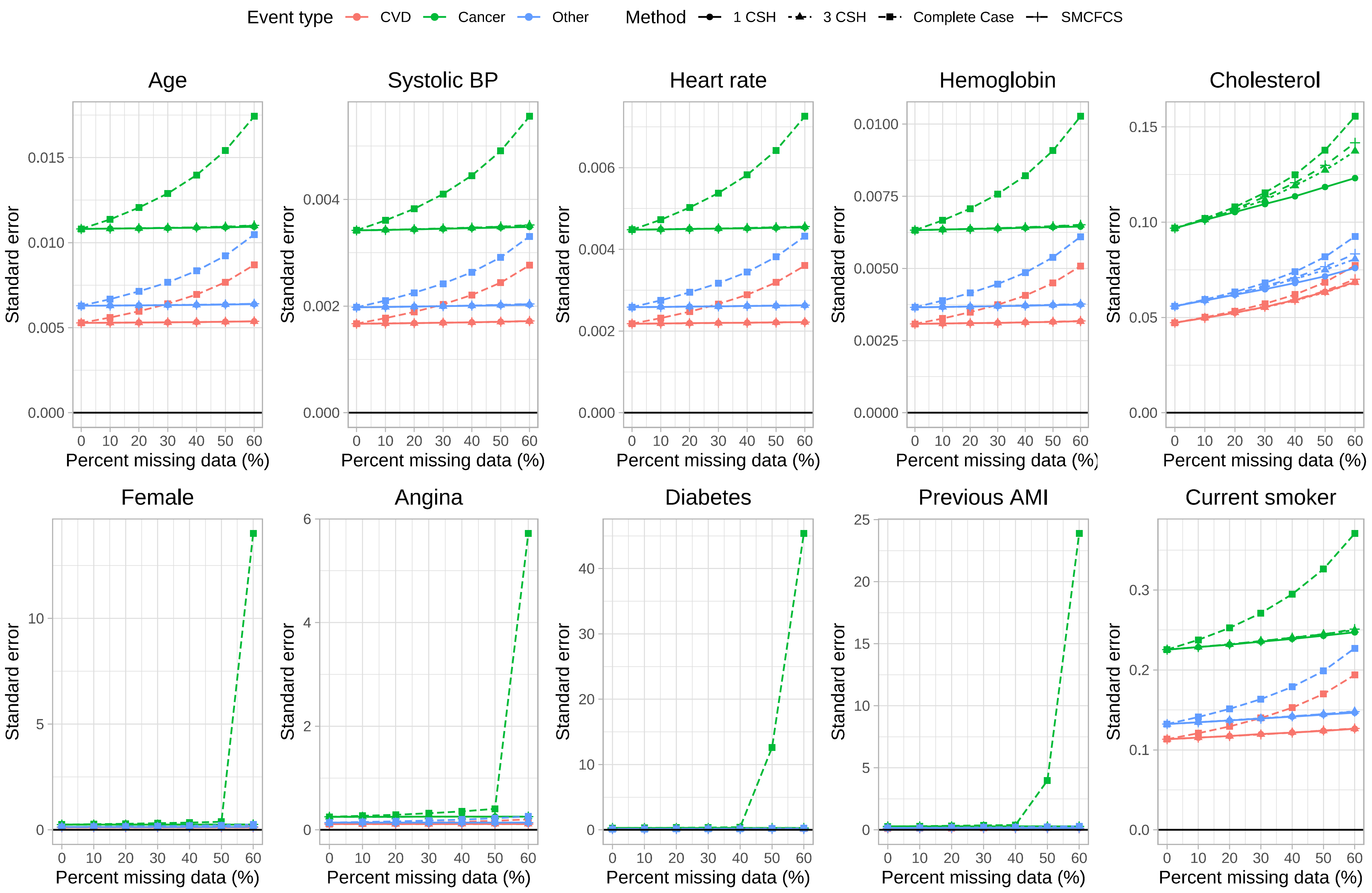

Figure A 14 . Mean standard error ($\lambda_{cvd} = 1$ & $\lambda_{cancer} = 2$ & $\lambda_{other} = 2$)

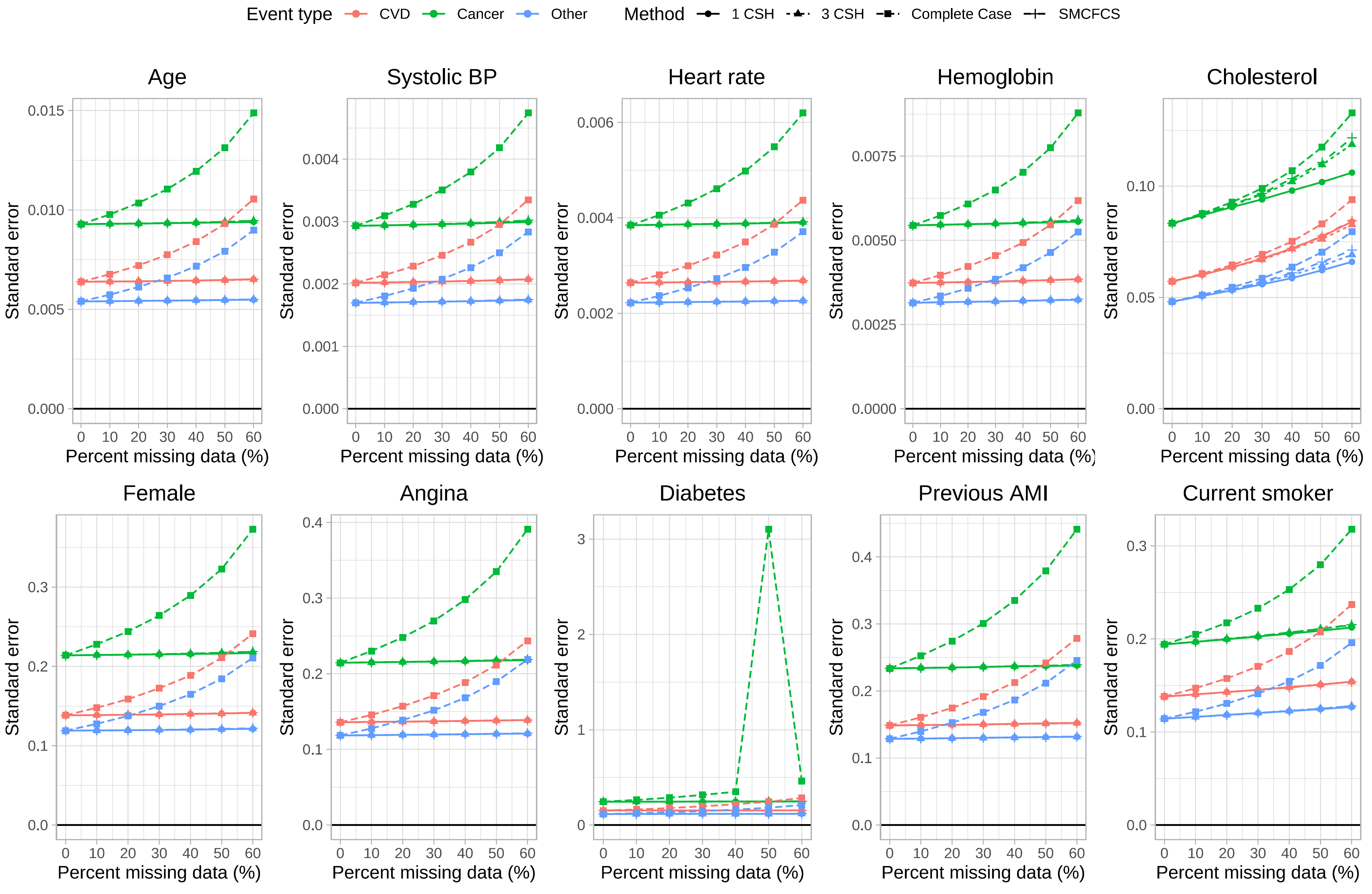

Figure A 15 . Mean standard error ($\lambda_{cvd} = 1$ & $\lambda_{cancer} = 3$ & $\lambda_{other} = 3$)

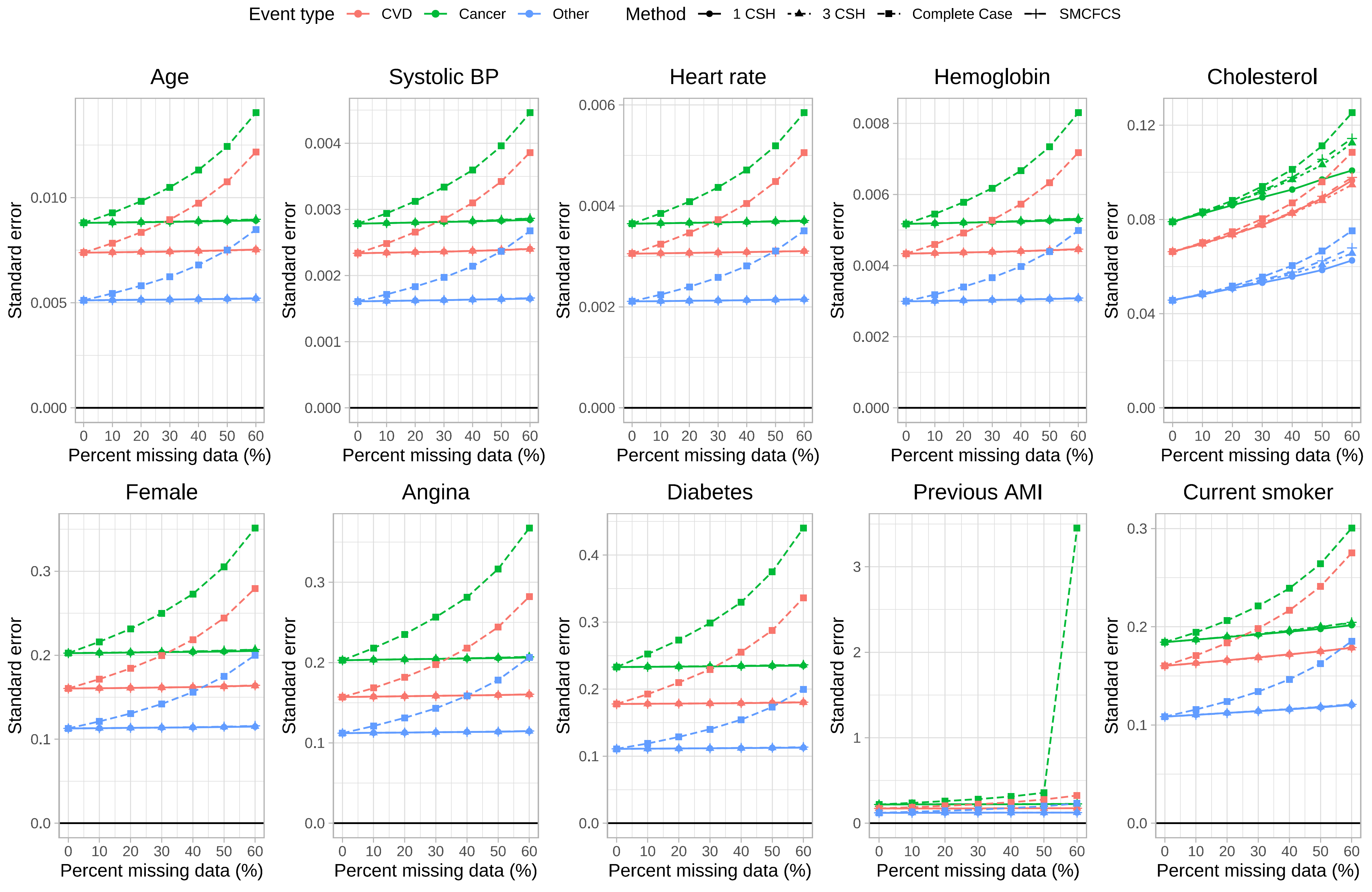

Supplement: Supplementary file 1 — (PDF 7247 kb) [file 180_2024_1518_MOESM1_ESM.pdf]
